# The Role of Vitamin D Supplementation in Type 1, Type 2, and Gestational Diabetes: A Comprehensive Updated Narrative Review

**DOI:** 10.3390/clinpract15080148

**Published:** 2025-08-07

**Authors:** Asala Nasser, Dimitrios Papandreou, Sousana K. Papadopoulou, Leila Cheikh Ismail

**Affiliations:** 1 Department of Clinical Nutrition, College of Health Sciences, University of Sharjah, Sharjah 27272, United Arab Emirates; u20104235@sharjah.ac.ae (A.N.); lcheikhismail@sharjah.ac.ae (L.C.I.); 2 Department of Nutritional Sciences and Dietetics, School of Health Sciences, International Hellenic University, 57400 Thessaloniki, Greece; sousana@ihu.gr

**Keywords:** vitamin D, diabetes type 1, diabetes type 2, gestational diabetes mellitus

## Abstract

Vitamin D has emerged as a modulatory factor in the pathogenesis and management of diabetes mellitus due to its influence on pancreatic β-cell function, immune regulation, and inflammatory pathways. This narrative review critically examines mechanistic and clinical evidence linking vitamin D status with type 1 diabetes (T1DM), type 2 diabetes (T2DM), and gestational diabetes (GDM). In T1DM, vitamin D’s immunomodulatory effects are thought to protect β-cells from autoimmune destruction; epidemiological studies associate vitamin D sufficiency with lower T1DM incidence and improved glycemic control, although causality remains under investigation. In T2DM, vitamin D deficiency is associated with worsened metabolic control and may contribute to disease development in at-risk individuals; however, it does not influence the initial onset of T2DM in patients who are already diagnosed. Intervention trials indicate that correcting the deficiency can modestly improve insulin sensitivity, β-cell function, and metabolic parameters. GDM has similarly been linked to hypovitaminosis D, with low maternal vitamin D levels associated with higher GDM risk and adverse perinatal outcomes; mechanistic insights suggest that adequate vitamin D supports glucose homeostasis in pregnancy, and emerging trials demonstrate improved insulin resistance with maternal vitamin D supplementation. Across these diabetes subtypes, maintaining sufficient vitamin D levels appears to confer metabolic benefits and may serve as an adjunct to current preventive and therapeutic strategies. However, definitive evidence from large-scale trials is required to establish optimal vitamin D supplementation protocols and confirm its efficacy in diabetes care.

## 1. Introduction

**Figure 1:**
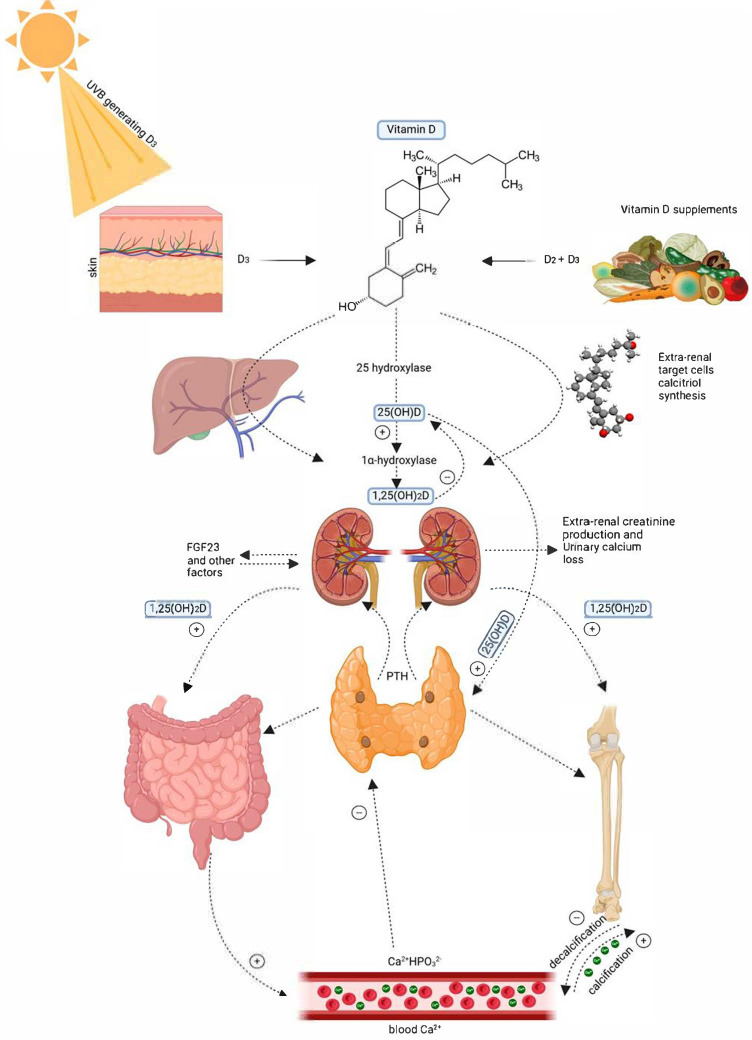
Metabolism of vitamin D. Adapted with permission from Voiculescu et al., 2025 [4].

Vitamin D is a fat-soluble hormone essential for maintaining calcium homeostasis and bone health. The human body can synthesize vitamin D upon skin exposure to ultraviolet B (UVB) radiation from sunlight [1]. The term “vitamin D” includes two main forms: vitamin D_2_ (ergocalciferol) and vitamin D_3_ (cholecalciferol). Ergocalciferol is derived from plant sterols (e.g., ergosterol) when exposed to UV light, whereas cholecalciferol is produced in the skin from 7-dehydrocholesterol via a UVB-dependent reaction [2]. Vitamin D_3_ is also obtained from animal-based foods (such as cod liver oil, fatty fish, and egg yolks) and fortified products [3]. Once synthesized in the skin or obtained from dietary sources, vitamin D is either sequestered in adipose tissue or transported to the liver, where it is converted into 25 hydroxyvitamin D [25(OH)D], the major circulating metabolite. In the kidney, 25(OH)D is further converted to 1,25 dihydroxyvitamin D, the hormonally active form that binds to vitamin D receptors (VDRs) and regulates gene expression in multiple tissues [1]. Figure 1 [4] provides a comprehensive diagram of vitamin D metabolism. Diabetes mellitus (DM) is a long-term metabolic condition characterized by consistently elevated blood glucose levels [5]. It has become a major global health concern, with projections indicating a 10.1% increase in the global prevalence of diabetes by 2030 [6]. By 2025, it is estimated that 380 million people worldwide may develop diabetes, including approximately 70 million in India (up from 41 million in 2007) [7]. It was also estimated that over 3.4 million people aged 20–79 died from diabetes-related causes in 2024 [8]. This rising burden underscores the importance of early detection and intervention in high-risk populations [9]. Gestational diabetes mellitus (GDM), on the other hand, arises due to pregnancy-induced hormonal changes—primarily, increases in placental hormones such as human placental lactogen and progesterone—that impair insulin action, resulting in insulin resistance. When pancreatic β-cells fail to compensate with adequate insulin secretion, hyperglycemia develops, typically manifesting in the second or third trimester. [10,11].

**Figure 2:**
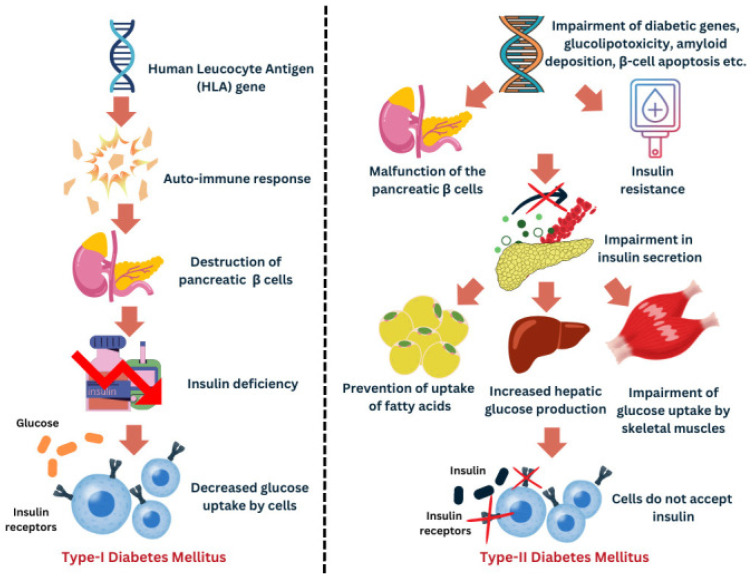
Comparative pathophysiology of T1DM and T2DM. Adapted with permission from Siam et al., 2024 [12].

There are multiple forms of diabetes, among which type 1, type 2, and gestational diabetes mellitus (Figure 2) [12] are prominent [8]. Type 2 diabetes (accounting for over 90% of cases) arises from insulin resistance coupled with an inadequate compensatory insulin secretion, often associated with obesity and a sedentary lifestyle. Type 1 diabetes (about 5–10% of cases) is an autoimmune disease in which the immune system destroys pancreatic β-cells, leading to absolute insulin deficiency [13]. Diabetes of any type can affect numerous organ systems and, therefore, remains a complex, ongoing public health challenge.

Despite extensive research, the precise etiology of diabetes is not fully understood. Chronic inflammation, oxidative stress, and immune dysfunction are thought to play key roles in its pathogenesis [14]. In recent years, vitamin D has garnered attention as a potential modifier of diabetes risk and progression. Vitamin D influences several pathways relevant to glycemic control; it regulates calcium homeostasis, which is important for insulin secretion [15], and it exerts antioxidant and immunomodulatory effects that could mitigate processes underlying diabetes [1]. Notably, vitamin D receptors are expressed in numerous tissues apart from bones, including pancreatic islet cells and various immune cells, suggesting that vitamin D may impact cell function and immune regulation in ways that influence diabetes development [16].

Given the escalating global prevalence of diabetes, there is an urgent need to identify novel preventive and therapeutic strategies. Lifestyle interventions can prevent the majority of type 2 diabetes cases; up to 90% have been attributed to modifiable behaviors [17], but maintaining long-term lifestyle changes is challenging. Moreover, modifiable risk factors for type 1 diabetes remain poorly understood. These gaps highlight the importance of exploring other factors, such as micronutrient status, that could be altered to improve outcomes. Emerging evidence from both animal models and human studies suggests that vitamin D status may influence diabetes risk [18]. Previous comprehensive reviews have also emphasized the role of vitamin D in the prevention and progression of both diabetes and cardiovascular disease, further highlighting the clinical relevance of this micronutrient in metabolic health [19]. This narrative review examines the current evidence on vitamin D supplementation in the context of type 1, type 2, and gestational diabetes mellitus, including its potential mechanisms of action, effects on risk and disease management, and implications for clinical practice.

## 2. Methodology

This narrative review was conducted following the SANRA 2.0 (Scale for the Assessment of Narrative Review Articles) recommendations for high-quality narrative reviews and the PRISMA-S extension for transparent reporting of literature searches, where applicable. The approach, however, did not involve a prospectively registered protocol, nor did it aim to pool results quantitatively in a meta-analysis. We systematically searched PubMed/MEDLINE, Scopus, and Google Scholar from January 2000 to June 2025 to capture contemporary mechanistic, clinical, and epidemiological evidence linking vitamin D with diabetes risk, progression, and management. Reference lists of all included reviews and eligible primary studies were hand-searched to identify additional relevant publications. The following concept blocks were combined with Boolean operators (“AND” between concepts; “OR” within concepts). Truncations and Medical Subject Headings (MeSH) were adapted to each database: Exposure/Intervention: “vitamin D” OR “cholecalciferol” OR “ergocalciferol” OR “25-hydroxyvitamin D” OR “1,25-dihydroxyvitamin D” OR “calcifediol” OR “calcitriol” OR “supplement*”. Condition: “diabetes” OR “type 1 diabetes” OR “T1DM” OR “type 2 diabetes” OR “T2DM” OR “gestational diabetes” OR “GDM” OR “prediabetes” OR “insulin resistance”. Study design filters (non-restrictive for narrative synthesis): “randomized controlled trial” OR “cohort” OR “case-control” OR “cross-sectional” OR “systematic review” OR “meta-analysis” OR “Mendelian randomization”.

Inclusion criteria included children, adolescents, adults, and pregnant women with or at risk of T1DM, T2DM, or GDM; any form, dose, and duration of vitamin D supplementation with outcomes; diabetes incidence, glycemic indices (e.g., HbA1c, FPG, 2h-OGTT, HOMA-IR, and insulin secretion/β-cell function), insulin dose requirements, inflammatory markers, cardiovascular/metabolic risk factors, and maternal–fetal outcomes (for GDM). Exclusion criteria included animal-only or in vitro studies; case reports/series, narrative commentaries without primary data, editorials, and conference abstracts without full data; studies where vitamin D was administered only as part of a multi-nutrient intervention without the ability to isolate vitamin D’s effect.

## 3. Mechanisms Linking Vitamin D with Diabetes Pathophysiology

Vitamin D’s role in glucose metabolism and immune modulation has been widely investigated, yet the mechanistic pathways remain only partially understood and are often derived from experimental or observational findings rather than robust causal evidence. The vitamin D receptor (VDR) is expressed in insulin-responsive and immune cells, including pancreatic β-cells, adipocytes, skeletal muscle, and T lymphocytes, suggesting a potential regulatory role in diabetes pathophysiology [14,15]. Activation of VDR in pancreatic β-cells modulates calcium-dependent insulin secretion, as vitamin D helps maintain the intracellular calcium levels necessary for insulin exocytosis [16,17]. While animal models and small-scale human studies support this pathway, its translational relevance remains unclear, and the impact of vitamin D on insulin secretion may be overshadowed by other metabolic stressors such as obesity, inflammation, and genetic predisposition. Another suggested mechanism involves enhanced peripheral insulin sensitivity through the upregulation of insulin receptor expression and improved glucose uptake in muscle and adipose tissues [18,19]. However, randomized clinical trials (RCTs) provide inconsistent evidence, with benefits often limited to vitamin-D-deficient individuals, indicating that supplementation may primarily correct deficiency rather than confer additional advantages beyond sufficiency. Vitamin D’s immunomodulatory effects, including the promotion of regulatory T-cell differentiation, suppression of pro-inflammatory cytokines (e.g., TNF-α and IL-6), and attenuation of dendritic cell activity, are biologically plausible mechanisms that may protect β-cells from autoimmune destruction in type 1 diabetes [20,21].

Despite these hypotheses, human RCTs have not conclusively demonstrated that vitamin D supplementation alone alters autoimmune progression or preserves β-cell function in clinical settings.

Overall, although vitamin D influences several biological pathways relevant to diabetes, current evidence is insufficient to confirm a direct and clinically meaningful causal effect. Many associations are likely confounded by factors such as sun exposure, physical activity, and general nutritional status. Well-designed mechanistic trials that integrate functional biomarkers of vitamin D action are needed to clarify its independent role in diabetes pathogenesis.

## 4. Results

### 4.1. Vitamin D and Type 1 Diabetes Mellitus

Type 1 diabetes mellitus (T1DM) is an autoimmune condition marked by the T-cell-driven destruction of insulin-producing β-cells in the pancreas, resulting in total insulin deficiency and a lifelong need for insulin replacement [13,22]. While genetic predisposition is a primary determinant of T1DM risk, environmental factors, potentially including vitamin D status, have been implicated in disease onset and progression [15,20]. The immunomodulatory properties of vitamin D suggest that sufficient vitamin D levels might help modulate the autoimmune processes underlying T1DM [23].

An increasing number of epidemiological studies suggest an association between vitamin D levels and the likelihood of developing type 1 diabetes mellitus (T1DM). A landmark birth-cohort study in Finland found that infants who received regular vitamin D supplementation had an approximately 80% lower risk of developing T1DM later in life [24]. A subsequent systematic review and meta-analysis similarly reported a 29% reduction in T1DM risk among children who were supplemented with vitamin D [25]. More recently, Munger et al. [26] observed that adults with higher serum 25(OH)D concentrations had a 44% lower relative risk of T1DM compared to those with lower vitamin D levels. Likewise, a 2021 meta-analysis identified vitamin D deficiency as a significant risk factor for T1DM [27]. In addition, a Mendelian randomization study by Manousaki et al. [28] provided genetic evidence supporting a causal link between lower vitamin D levels and higher risk of T1DM, reinforcing the biological plausibility of this association. Similarly, the TRIGR nested case-control ancillary study by Miettinen et al. [29] found that lower serum 25(OH)D levels during early childhood were associated with increased risk of islet autoimmunity and progression to T1DM. Together, these findings suggest that adequate vitamin D exposure, particularly early in life, may have a protective effect against the development of T1DM. Among individuals with established T1DM, vitamin D status has been correlated with disease control. Vitamin D deficiency is common in T1DM and has been associated with suboptimal glycemic control. Alharbi et al. [30] reported that 52.5% of children with T1DM in Saudi Arabia were vitamin D deficient, and those deficient had significantly higher glycated hemoglobin (HbA1c) levels, indicating worse blood glucose control. Similarly, Gabbay et al. [27] found that T1DM patients with vitamin D deficiency required higher insulin doses and had higher HbA1c compared to patients with sufficient vitamin D.

Emerging evidence suggests that correcting vitamin D deficiency may improve metabolic control in T1DM. Some interventional studies have noted that vitamin D supplementation can reduce insulin requirements and lower HbA1c in vitamin-D-deficient T1DM patients [31]. These findings imply that vitamin D status is an important factor in T1DM management and that supplementation could be a beneficial adjunct therapy, particularly for those with deficiency [22]. Mechanistic studies provide biological plausibility for these clinical observations. Vitamin D influences immune function, for instance, by promoting regulatory T cells and diminishing pro-inflammatory responses, which could help preserve β-cell function in an autoimmune context [21]. This offers a plausible explanation for vitamin D’s protective associations in T1DM. Interventional trials of vitamin D supplementation in T1DM have yielded mixed results. For example, Gabbay et al. [27] supplemented T1DM patients who were vitamin D deficient with 2000 IU of cholecalciferol daily, and after one year, the treated group showed improved HbA1c levels and reduced insulin requirements compared to baseline. However, not all studies have demonstrated such benefits, likely due to variations in vitamin D dosage, the duration of therapy, and patient characteristics [20].

Recent systematic reviews and meta-analyses provide further insight into vitamin D’s role in T1DM. Dong et al. [20] and Chen et al. [32] both reported that vitamin D supplementation was associated with modest improvements in glycemic control (including reductions in HbA1c and fasting glucose) in patients with T1DM, especially among those with low baseline vitamin D levels. These findings suggest that vitamin D supplementation can be a useful adjunct in managing T1DM, although it is not a replacement for standard insulin therapy. Numerous observational studies also consistently link higher vitamin D status to a lower incidence of T1DM and to improved glycemic control in those already affected [31,33]. Ensuring adequate vitamin D levels from early life onward may be a cost-effective strategy to help reduce the burden of T1DM. Nevertheless, further large-scale randomized trials are needed to determine optimal vitamin D dosing protocols and to identify the patients who would derive the greatest benefit from supplementation in the context of T1DM. Overall, the evidence indicates that vitamin D plays a notable role in T1DM. Table 1 summarizes RCTs, clinical, epidemiological, and observational studies, and systematic reviews.

### 4.2. Vitamin D and Type 2 Diabetes Mellitus

Type 2 diabetes mellitus (T2DM) is a metabolic disease defined by insulin resistance accompanied by progressive β-cell dysfunction, leading to chronic hyperglycemia [19,35]. While genetic susceptibility, obesity, and physical inactivity are primary contributors to its development, there is growing interest in the contribution of micronutrient status. In particular, vitamin D deficiency has emerged as a potentially modifiable risk factor for T2DM [19]. As the prevalence of T2DM continues to rise worldwide, addressing vitamin D deficiency, an easily measurable and correctable condition, could become a crucial component of prevention and management strategies [36]. Epidemiological studies have consistently shown an association between low vitamin D status and an increased risk of T2DM. For instance, in a large Danish cohort, individuals with vitamin D deficiency had a significantly higher incidence of type 2 diabetes, particularly among younger adults [37]. Additionally, Charoenngam and Holick [24] reviewed evidence showing that vitamin D deficiency correlates with greater insulin resistance and impaired β-cell function, conditions that precede and exacerbate T2DM. These findings suggest that inadequate vitamin D levels contribute to the progression of insulin resistance and may influence the onset of T2DM [35]. Mechanistic studies support the link that vitamin D is signaling in pancreatic and muscle cells and has been shown to enhance insulin release while tempering inflammation, providing a biological basis for the observed epidemiological associations [18]. This is particularly relevant considering the established role of vitamin D in modulating adipogenesis and inflammatory pathways in obesity-related insulin resistance [32]. Clinical trials assessing vitamin D supplementation for T2DM prevention or management have produced mixed results. The landmark D2d trial conducted by Pittas et al. [38] evaluated 4000 IU/day of vitamin D_3_ in individuals with prediabetes over 2.5 years and found no significant reduction in diabetes incidence for the overall population. However, in secondary analyses of that trial, participants with severe vitamin D deficiency at baseline or those who achieved the highest vitamin D levels with supplementation did exhibit a reduced rate of progression to diabetes, suggesting that vitamin D’s efficacy may depend on baseline status [19,35]. This conclusion was further supported by a 2023 systematic review and meta-analysis by Farahmand et al. [39], which found that vitamin D supplementation significantly improved glycemic indices—including fasting plasma glucose, HbA1c, and HOMA-IR—in individuals with type 2 diabetes, particularly those with pre-existing vitamin D deficiency. Additionally, an umbrella review by Cheng et al. (2024) [40] consolidated findings from multiple meta-analyses and confirmed that vitamin D supplementation plays both a preventive and therapeutic role in T2DM, especially when appropriate dosing and patient selection are applied. In addition, a 2022 meta-analysis by Dong et al. reported modest improvements in glycemic indices (fasting plasma glucose, HOMA-IR insulin resistance, and HbA1c) with vitamin D supplementation, especially among individuals who were vitamin D deficient before intervention [14]. Another recent meta-analysis [34] similarly noted significant reductions in HbA1c and fasting glucose in T2DM patients receiving vitamin D. These findings suggest that while vitamin D supplementation may not universally prevent T2DM, it can confer metabolic benefits in vitamin-D-deficient populations. Table 2 summarizes, RCTs, clinical, epidemiological and observational studies as well as systematic reviews. Beyond glycemic parameters, vitamin D supplementation might influence other aspects of T2DM comorbidity. Nascimento et al. [36] observed that vitamin D supplementation was associated with small but favorable changes in cardiovascular risk markers, including reductions in total cholesterol, low-density lipoprotein (LDL) cholesterol, and blood pressure. Improvements in the inflammatory profile have also been documented; for example, vitamin D supplementation has been shown to decrease circulating pro-inflammatory cytokines and to increase adiponectin levels, changes that could further enhance insulin sensitivity [14,18]. Such pleiotropic effects of vitamin D are particularly relevant given the heightened cardiovascular risk in individuals with T2DM.

There is substantial heterogeneity across trials in terms of vitamin D dosing regimens, duration of supplementation, baseline vitamin D status of participants, and outcome measures, which makes it challenging to draw firm conclusions [35]. Many studies have relatively short follow-up periods, limiting insight into the long-term effects of supplementation [36]. Furthermore, confounding factors such as dietary vitamin D intake, sunlight exposure, and adherence to supplementation were not always adequately controlled, potentially influencing results [30,35]. These factors may partly explain why some studies fail to show significant benefits of vitamin D in T2DM.

To conclude, accumulating evidence supports a contributory role for vitamin D in T2DM prevention and management. Sufficient vitamin D availability appears to promote insulin secretion, improve insulin sensitivity, and reduce inflammatory mediators [19]. While vitamin D supplementation is not a substitute for standard therapies in diabetes, correcting vitamin D deficiency, particularly in high-risk or deficient individuals, could be a simple and safe adjunctive strategy to improve metabolic outcomes. Further long-term, well-powered trials are needed to establish optimal dosing strategies and to conclusively identify which subsets of patients benefit most from vitamin D supplementation in T2DM.

### 4.3. Vitamin D and Gestational Diabetes Mellitus

Gestational diabetes mellitus (GDM) refers to impaired glucose tolerance that is initially identified during pregnancy. It presents considerable health risks for both the expectant mother and the developing fetus. Affected pregnancies have higher rates of maternal complications such as gestational hypertension and preeclampsia, an increased likelihood of cesarean delivery, and a greater long-term risk of the mother developing type 2 diabetes post-pregnancy [15,41,42]. Infants born to mothers with GDM are more prone to macrosomia, neonatal hypoglycemia, and a higher risk of obesity or glucose intolerance later in life [43]. Given the serious health implications of GDM, there has been growing interest in identifying modifiable risk factors, including nutritional factors such as vitamin D status. It has been hypothesized that ensuring adequate vitamin D during pregnancy might improve maternal glucose metabolism and thereby reduce the risk of GDM [41].

Several studies have consistently shown an association between low maternal vitamin D levels and an increased risk of developing GDM. In a meta-analysis of 24 observational studies, Zhang et al. [22] found that pregnant women with vitamin D deficiency had a significantly higher risk of GDM compared to those with sufficient vitamin D levels. Similarly, a prospective study by Bi et al. [21] reported that women with insufficient 25(OH)D, particularly in early pregnancy, were more likely to develop impaired glucose tolerance and subsequent GDM. Parlea et al. [23] also demonstrated that lower serum vitamin D concentrations in early gestation were associated with an increased incidence of GDM later in pregnancy. These findings, echoed by other studies, indicate a clear pattern: vitamin D insufficiency in pregnancy correlates with elevated GDM risk [43,44]. Nevertheless, these observational associations do not confirm a causal relationship. Low vitamin D status could be a marker for other factors (such as higher adiposity, certain ethnic backgrounds, or reduced sun exposure) that themselves predispose to GDM [27,42]. Rigorous studies are needed to isolate the effect of vitamin D from such confounders. There is also a strong biological plausibility for vitamin D’s influence on GDM development. VDRs are present in tissues central to glucose regulation, including pancreatic β-cells and skeletal muscle [42]. Vitamin D can enhance pancreatic insulin secretion by modulating β-cell calcium levels, and it may improve insulin sensitivity by reducing circulating pro-inflammatory cytokines that impair insulin action [17]. These mechanisms support the hypothesis that sufficient vitamin D status could improve maternal glucose homeostasis and thereby lower GDM risk [43]. Consistent with this hypothesis, both observational and laboratory studies have reinforced a link between vitamin D and glucose metabolism in pregnancy [41,44].

Intervention studies in pregnant women have begun to explore whether vitamin D supplementation can prevent GDM or improve metabolic markers during pregnancy. In a randomized trial, [44] observed that higher doses of vitamin D, 50,000 IU every two weeks, led to improved insulin sensitivity and lower fasting glucose levels in pregnant women, compared to standard prenatal care. In a systematic review and meta-analysis, Poel et al. [45] concluded that vitamin D supplementation had a modest beneficial effect on insulin resistance in pregnancy, although results across studies were variable. Harvey et al. [46] similarly noted that vitamin D supplementation may improve some maternal metabolic outcomes, but they also highlighted inconsistencies among trials. Furthermore, Corcoy et al. [47] Overall, while vitamin D supplementation appears to positively influence glycemic measures in pregnancy, the magnitude of effect has been modest, and study designs have varied considerably. Table 3 summarizes all intervention, observational, and systematic review studies.

Besides glucose metabolism, maternal vitamin D status has been linked to other pregnancy outcomes. In a comprehensive review, Harvey et al. [46] reported that pregnant women with insufficient vitamin D had higher rates of preeclampsia, low-birth-weight infants, and preterm delivery compared to those with adequate vitamin D levels. Another analysis by Bi et al. [21] found associations between maternal vitamin D deficiency and impaired fetal growth, as well as increased neonatal morbidity and mortality. Poel et al. [45] likewise observed that vitamin D insufficiency was tied to a range of adverse outcomes for both mother and baby. While these associations do not prove that vitamin D deficiency is the direct cause, they underscore the potential broader benefits of maintaining adequate vitamin D levels during pregnancy for maternal health and neonatal well-being.

Despite these encouraging findings, significant knowledge gaps persist regarding vitamin D and GDM. Most evidence to date is derived from observational studies, which are subject to confounding and cannot establish causality [27]. The available intervention trials are heterogeneous with respect to vitamin D dosage, timing of supplementation (early vs. late pregnancy), and outcome measures, making it challenging to determine the optimal approach [42,43]. Additionally, trial results have been inconsistent, partly due to varying study quality and sample sizes. In a systematic review of 11 studies [50], it was concluded that there is no moderate or high-quality evidence to support the efficacy of vitamin D supplementation in improving maternal and neonatal outcomes in pregnant women with GDM. While some secondary outcomes showed potential benefits, the overall quality of evidence was insufficient to draw definitive conclusions. The authors emphasized the need for further high-quality RCTs to better understand the role of vitamin D supplementation in this population. These issues highlight the need for rigorously designed, large-scale randomized controlled trials to clarify the role of vitamin D supplementation in GDM prevention and to develop evidence-based guidelines.

Overall, vitamin D deficiency in pregnancy has been consistently associated with an elevated risk of GDM, and vitamin D supplementation shows potential benefits for improving insulin sensitivity and glycemic control during gestation [27]. Current evidence is not yet sufficient to recommend universal high-dose vitamin D supplementation solely for GDM prevention. However, ensuring that pregnant women achieve adequate vitamin D status is a prudent measure given its potential benefits for metabolic health and its general safety. Future well-powered RCTs will be crucial to determine definitive supplementation guidelines and to identify those pregnant populations who may benefit most from vitamin D interventions.

## 5. Discussion

Taken together, the evidence reviewed above indicates that vitamin D status is an important factor in all three major forms of diabetes. Observational studies across type 1, type 2, and gestational diabetes populations consistently show that low vitamin D status is linked to higher disease risk or poorer metabolic outcomes [19,27,37]. These relationships are supported by plausible biological mechanisms: vitamin D influences both insulin secretion and insulin sensitivity, and it modulates immune responses that can affect autoimmune diabetes [18]. This convergence of epidemiological and mechanistic evidence suggests that vitamin D deficiency is not merely a consequence of ill health but may actively contribute to the pathophysiology of diabetes [35]. Despite these associations, randomized trials have yielded more nuanced results. Vitamin D supplementation by itself has not proven to be a silver bullet for preventing diabetes; rather, its benefits seem to depend on context. In type 1 diabetes, early-life supplementation (during infancy) was associated with a significantly reduced incidence, pointing to a potential critical window for vitamin D’s protective effect [24]. In established T1DM, supplementation appears to help mainly when there is an existing deficiency, and it may slow autoimmune destruction if initiated early in the disease course [22].

In type 2 diabetes or those at high risk for it, vitamin D supplementation shows modest improvements in glycemic control and metabolic parameters, predominantly in individuals with baseline vitamin D deficiency [34,36]. Those with sufficient levels do not seem to gain substantial benefit from additional vitamin D [35]. Similarly, in pregnancy, correcting vitamin D insufficiency may improve insulin sensitivity and certain pregnancy outcomes, but routine high-dose supplementation in all pregnant women has not yet demonstrated clear efficacy in preventing GDM [51]. These nuances underscore that vitamin D’s impact is greatest as a corrective measure for deficiency, rather than as an enhancement beyond normal vitamin D status. From a clinical standpoint, maintaining adequate vitamin D levels should be considered part of comprehensive care in populations at risk for diabetes. Ensuring vitamin D sufficiency through safe sun exposure, diet, or supplements per current guidelines is a low-cost, low-risk intervention with potential to improve metabolic health [1,3]. However, current evidence does not warrant recommending vitamin D supplements at pharmacological doses solely for diabetes prevention in individuals who are already vitamin D replete [35,36].

Future research should focus on filling the remaining gaps: well-designed, large-scale trials are needed to establish whether optimizing vitamin D status can directly lower the incidence of type 1 diabetes in genetically susceptible children, prevent type 2 diabetes in high-risk adults, or reduce the occurrence of GDM and its complications. Such trials should also clarify the optimal serum 25(OH)D thresholds for metabolic benefits, appropriate dosing regimens, and the timing of intervention (e.g., early life, pre-pregnancy, or early gestation) required to achieve meaningful outcomes [32].

Growing evidence emphasizes the crucial role of adequate vitamin D levels in metabolic function. Karras et al. [52], for instance, reported that vitamin D deficiency during pregnancy is linked to elevated insulin resistance, pointing to a possible involvement of vitamin D in regulating glucose metabolism in this group. Furthermore, Pludowski et al. [53] advocate for a daily supplementation of 2000 IU of vitamin D in adults to achieve optimal serum concentrations, which may contribute to improved metabolic outcomes, including reduced risk of type 2 diabete+s. In the meantime, it remains prudent for healthcare providers to monitor and correct vitamin D deficiency as part of overall health management for patients with or at risk of diabetes, given the broad health benefits of vitamin D and the minimal downsides of maintaining.

## 6. Strengths and Limitations

The present narrative review is distinguished by its comprehensive scope and integrative approach, covering all major forms of diabetes (Type 1, Type 2, and Gestational) and incorporating evidence from a broad spectrum of study designs. We synthesize findings from randomized controlled trials, meta-analyses, and observational studies to provide a nuanced understanding of vitamin D’s role in diabetes, and they articulate mechanistic pathways with exceptional clarity. This clear discussion of underlying biological mechanisms, such as vitamin D’s influence on β-cell function, insulin sensitivity, and immune modulation, not only strengthens the linkage between vitamin D and diabetic outcomes but also helps contextualize the diverse clinical findings. However, as an updated narrative review that relies on secondary data, the work is inherently limited by the quality and scope of available studies, which may introduce bias or heterogeneity into the conclusions. The review does not present original experimental data and forgoes a quantitative meta-analysis, meaning that it cannot offer new statistical effect size estimates or definitively resolve inconsistencies across studies. Despite these limitations, the paper’s breadth of coverage and critical analysis serve as significant strengths, enabling it to concisely synthesize current knowledge and highlight key insights and gaps in the literature.

## 7. Conclusions

Vitamin D has emerged as a noteworthy factor in the context of diabetes mellitus. This review highlights that vitamin D deficiency is common in individuals with type 1, type 2, and gestational diabetes and is associated with higher risks and poorer glycemic outcomes. Conversely, sufficient vitamin D levels achieved through sunlight exposure, diet, or supplementation correlate with lower incidence of type 1 diabetes, improved insulin sensitivity in type 2 diabetes, and possibly a reduced risk of GDM and its complications. The biological mechanisms underpinning these observations are well-supported, involving vitamin D’s roles in insulin secretion, glucose metabolism, and immune regulation. Vitamin D supplementation should thus be viewed as a supportive strategy alongside standard diabetes prevention and treatment measures, rather than a standalone therapy. Ensuring adequate vitamin D status, especially in high-risk groups such as individuals with prediabetes, patients with T1DM, or pregnant women, is a sensible public health goal given its general health benefits. At the same time, more research is needed to establish definitive guidelines for vitamin D supplementation in the context of diabetes care. Ongoing and future large-scale studies will help determine whether routine vitamin D optimization can effectively reduce the burden of diabetes and inform evidence-based recommendations for clinical practice.

## Figures and Tables

**Table 1:** Summary of RCTs, clinical, epidemiological, observational, and systematic review studies.

Study	Design	Population	Vitamin D Dose	Duration	Outcomes	Conclusion
Gabbay et al. [27]	Randomized Controlled Trial	T1DM patients with vitamin D deficiency	2000 IU/day cholecalciferol	1 year	Improved HbA1c, reduced insulin requirements	Vitamin D supplementation improved metabolic control in deficient patients
Dong et al. [20]	Meta-analysis of RCTs	T1DM patients	Varied	Varied	Modest reductions in HbA1c and fasting glucose	More effective in individuals with low baseline vitamin D
Chen et al. [32]	Meta-analysis of RCTs	T1DM patients	Varied	Varied	Improved glycemic control	Supports adjunctive role of vitamin D in T1DM management
Ferrari and Locatelli [25]	Systematic Review	Individuals with or at risk for T1DM	Varied	Varied	Reviewed immune modulation and metabolic effects of vitamin D	Vitamin D may preserve β-cell function and improve glycemic outcomes
Hyppönen et al. [34]	Birth Cohort Study (Epidemiological)	Finnish infants followed into adulthood	Regular supplementation (infancy)	Long-term follow-up	~80% reduced T1DM risk in those supplemented with vitamin D	Strong inverse association between early vitamin D and T1DM risk
Munger et al. [26]	Epidemiological Cohort Study	Adults with varied serum 25(OH)D levels	N/A (observational serum levels)	Not specified	Higher serum vitamin D linked with 44% lower T1DM risk	Supports protective effect of higher vitamin D status
Littorin et al. [35]	Case-control nested in population study	Newly diagnosed T1D patients (15–34 yrs, n=459) vs. matched controls (n=208), Sweden	No supplementation; 25OHD measured	At diagnosis + 8-year follow-up	T1D group had significantly lower 25OHD at diagnosis; further decline over 8 year	Low vitamin D levels at diagnosis may be linked to T1D development, especially in males.
Zipitis and Akobeng [33]	Systematic Review of Observational Studies	Children receiving vitamin D supplementation	Varied	Childhood	29% reduced risk of T1DM with vitamin D supplementation	Observational evidence supports protective role of vitamin D
Nascimento et al. [36]	Systematic review	Children and adolescents with type 1 diabetes (8 studies included)	Various doses across studies	2–12 months	Mixed results; some studies showed improved HbA1c, others no significant effect	Vitamin D may help improve glycemic control, but evidence remains inconsistent.
Manousaki et al., 2021 [28]	Mendelian Randomization Study	General population (T1DM genetics)	N/A	N/A	Low genetically predicted vitamin D ↑ T1DM risk	Genetic evidence for causal relationship
Miettinen et al.(TRIGR), 2020 [29]	Nested Case-Control (TRIGR)	Children at risk of T1DM	Serum 25(OH)D levels	childhood	No significant association	Vitamin D status in childhood not strongly linked to T1DM

**Table 2:** Summary of RCTs, clinical, epidemiological, observational, and systematic review studies.

Study	Design	Population	Vitamin D Dose	Duration	Outcomes	Conclusion
Pittas et al. [38]	Randomized Controlled Trial (D2d study)	Adults with prediabetes	4000 IU/day	2.5 years	No significant reduction in diabetes incidence overall; benefit seen in vitamin-D-deficient subgroup	Efficacy may depend on baseline vitamin D status
Dong et al. [20]	Meta-analysis of RCTs	T2DM patients	Varied	Varied	Modest improvements in FPG, HOMA-IR, HbA1c (mainly in deficient individuals)	Supplementation improves metabolic indices in vitamin-D-deficient patients
Chen et al. [32]	Meta-analysis of RCTs	T2DM patients	Varied	Varied	Reductions in HbA1c and fasting glucose	Supports beneficial effect of vitamin D on glycemic control
Barbarawi et al. [41]	Meta-analysis of RCTs	T2DM patients	Varied	Varied	Improved lipid profile, reduced BP, reduced inflammatory markers	Vitamin D may improve cardiometabolic outcomes
Mai et al. [17]	Interventional study	Adults with severe obesity (n = 48)	Single high dose (600,000 IU cholecalciferol)	1 month	Increased total and HMW adiponectin after supplementation	High-dose vitamin D_3_ improved adiponectin profile in obese patients.
Vojdeman et al. [42]	Observational Cohort Study	Danish adults	N/A	Not specified	Vitamin D deficiency associated with increased T2DM risk, especially in younger adults	Supports a link between low vitamin D and increased diabetes incidence
Pittas et al., 2019 [38]	RCT	Adults at high risk of T2DM	4000 IU/day cholecalciferol	Median 2.5 years	No significant ↓ in diabetes incidence overall	Subgroup benefit in those with low vitamin D
Farahmand et al., 2023 [39]	Systematic Review and Meta-analysis	People with T2DM	Varied	Varied	↓ HbA1c, ↓ fasting glucose, ↓ insulin resistance	Supports glycemic control in T2DM
Cheng et al., 2024 [40]	Umbrella Review	Mixed (T2DM, cohort + RCTs)	Varied	Varied	Improved control and reduced T2DM risk	Strong evidence for benefit in T2DM

**Table 3:** Summary of RCTs, observational, and systematic review studies.

Study	Design	Population	Vitamin D Dose	Timing	Outcomes	Conclusion
Soheilykhah et al. [48]	RCT	Pregnant women	50,000 IU every two weeks	During pregnancy	Improved insulin sensitivity, reduced fasting glucose levels	Higher dose vitamin D may improve glycemic control
Corcoy et al. [47]	RCT	Pregnant Women	1600 IU daily	During Pregnancy	Improved Vitamin D sufficiency	Small statistically reduction in FBG-No effect on insulin
Poel et al. [45]	Systematic Review and Meta-analysis of RCTs	Pregnant women across multiple studies	Varied	Varied	Modest improvement in insulin resistance	Vitamin D may improve insulin sensitivity; results variable
Harvey et al. [46]	Systematic Review (includes RCTs)	Pregnant women	Varied across studies	Varied	Possible improvements in metabolic markers	Inconsistencies in trial findings noted
Littorin et al. [35]	Case-control nested in population study	Newly diagnosed T1D patients (15–34 yrs, n = 459) vs. matched controls (n = 208), Sweden	No supplementation; 25OHD measured	At diagnosis + 8-year follow-up	T1D group had significantly lower 25OHD at diagnosis; further decline over 8 year	Low vitamin D levels at diagnosis may be linked to T1D development, especially in males.
Kron-Rodrigues et al. [49]	Systematic Review and meta-analysis of 11 studies	Pregnant women	Varied	Varied	No differences in the frequency of cesarean deliveries	No high-quality evidence to support vitamin D supplementation
Bi et al. [21]	Prospective Observational Study	Pregnant women	N/A	Early pregnancy	Insufficient vitamin D linked to impaired glucose tolerance and GDM	Early deficiency may increase GDM risk
Parlea et al. [23]	Observational Cohort	Pregnant women	N/A	Early pregnancy	Low 25(OH)D associated with higher GDM incidence later in pregnancy	Low vitamin D status may contribute to GDM
Buchanan and Xiang, 2005 [10]	Review Study	Women with Gestational Diabetes	N/A	N/A	Explores pathogenesis and consequences	Summarizes GDM as predictor of future diabetes risk
Buchanan et al. [11]	Narrative review	Pregnant women with/at risk of gestational diabetes	N/A	N/A	Explores complex pathophysiology beyond glucose intolerance	GDM is a heterogeneous condition with diverse underlying mechanisms

## Data Availability

No new data were created or analyzed in this study. Data sharing is not applicable to this article.

## References

[1] Holick M.F. (2007). Vitamin D deficiency. N. Engl. J. Med..

[2] (2022). D Fact Sheet for Health Professionals. https://ods.od.nih.gov/factsheets/VitaminD-HealthProfessional/.

[3] Ross A.C., Taylor C.L., Yaktine A.L., Del Valle H.B. (2011). Dietary Reference Intakes for Calcium and Vitamin D.

[4] Voiculescu V.M., Nelson Twakor A., Jerpelea N., Pantea Stoian A. (2025). Vitamin D: Beyond Traditional Roles—Insights into Its Biochemical Pathways and Physiological Impacts. Nutrients.

[5] Wild S., Roglic G., Green A., Sicree R., King H. (2004). Global prevalence of diabetes: Estimates for the year 2000 and projections for 2030. Diabetes Care.

[6] Shaw J.E., Sicree R.A., Zimmet P.Z. (2010). Global estimates of the prevalence of diabetes for 2010 and 2030. Diabetes Res. Clin. Pract..

[7] Mohan V., Sandeep S., Deepa R., Shah B., Varghese C. (2007). Epidemiology of type 2 diabetes: Indian scenario. Indian J. Med. Res..

[8] International Diabetes Federation (2025). IDF Diabetes Atlas.

[9] American Diabetes Association (2023). Classification and diagnosis of diabetes: Standards of Medical Care in Diabetes—2023. Diabetes Care.

[10] Buchanan T.A., Xiang A.H. (2005). Gestational diabetes mellitus. J. Clin. Investig..

[11] Buchanan T.A., Xiang A.H., Page K.A., Watanabe R.M. (2025). What is Gestational Diabetes—Really?. Diabetes.

[12] Siam N.H., Snigdha N.N., Tabasumma N., Parvin I. (2024). Diabetes Mellitus and Cardiovascular Disease: Exploring Epidemiology and Pathophysiology. Rev. Cardiovasc. Med..

[13] Atkinson M.A., Eisenbarth G.S., Michels A.W. (2014). Type 1 diabetes. Lancet.

[14] Wellen K.E., Hotamisligil G.S. (2005). Inflammation, stress, and diabetes. J. Clin. Investig..

[15] Chiu K.C., Chu A., Go V.L., Saad M.F. (2004). Hypovitaminosis D is associated with insulin resistance and beta cell dysfunction. Am. J. Clin. Nutr..

[16] Norman A.W. (2008). From vitamin D to hormone D: Fundamentals of the vitamin D endocrine system essential for good health. Am. J. Clin. Nutr..

[17] Mai S., Walker G.E., Vietti R., Cattaldo S., Mele C., Priano L., Mauro A., Bona G., Aimaretti G., Scacchi M. (2017). Acute Vitamin D_3_ supplementation in severe obesity: Evaluation of multimeric adiponectin. Nutrients.

[18] Pittas A.G., Lau J., Hu F.B., Dawson-Hughes B. (2007). The role of vitamin D and calcium in type 2 diabetes. A systematic review and meta-analysis. J. Clin. Endocrinol. Metab..

[19] Papandreou D., Hamid Z. (2015). The role of vitamin D in diabetes and cardiovascular disease: An updated review of the literature. Dis. Markers.

[20] Dong J.Y., Zhang W.G., Chen J.J., Zhang Z.L., Han S.F., Qin L.Q. (2013). Vitamin D intake and risk of type 1 diabetes: A meta-analysis of observational studies. Nutrients.

[21] Bi W.G., Nuyt A.M., Weiler H., Leduc L., Santamaria C., Wei S.Q. (2018). Association between vitamin D supplementation during pregnancy and offspring growth, morbidity, and mortality: A systematic review and meta-analysis. JAMA Pediatr..

[22] Zhang M.X., Pan G.T., Guo J.F., Li B.Y., Qin L.Q., Zhang Z.L. (2015). Vitamin D deficiency increases the risk of gestational diabetes mellitus: A meta-analysis of observational studies. Nutrients.

[23] Parlea L., Bromberg I.L., Feig D.S., Vieth R., Merman E., Lipscombe L.L. (2012). Association between serum 25-hydroxyvitamin D in early pregnancy and risk of gestational diabetes mellitus. Diabet. Med..

[24] Charoenngam N., Holick M.F. (2022). Immunologic effects of vitamin D on human health and disease. Nutrients.

[25] Ferrari D., Locatelli M. (2022). Vitamin D and type 1 diabetes mellitus: State of the art. Trends Endocrinol. Metab..

[26] Munger K.L., Levin L.I., Hollis B.W., Howard N.S., Ascherio A. (2013). Serum 25-hydroxyvitamin D levels and risk of type 1 diabetes in young adults. Am. J. Epidemiol..

[27] Gabbay M.A., Sato M.N., Finazzo C., Duarte A.J., Dib S.A. (2012). Effect of cholecalciferol as adjunctive therapy with insulin on protective immunologic profile and decline of residual β-cell function in new-onset type 1 diabetes mellitus. Arch. Pediatr. Adolesc. Med..

[28] Manousaki D., Harroud A., Mitchell R.E., Ross S., Forgetta V., Timpson N.J., Smith G.D., Polychronakos C., Richards J.B. (2021). Vitamin D levels and risk of type 1 diabetes: A Mendelian randomization study. PLoS Med..

[29] Miettinen M.E., Niinistö S., Erlund I., Cuthbertson D., Nucci A.M., Honkanen J., Vaarala O., Hyöty H., Krischer J.P., Knip M. (2020). Serum 25-hydroxyvitamin D concentration in childhood and risk of islet autoimmunity and type 1 diabetes: The TRIGR nested case-control ancillary study. Diabetologia.

[30] Alharbi K.K., Khan I.A., Khan N., Alharbi F.K., Alghamdi J., Alshahrani M.Y., Alharbi K.F. (2022). Vitamin D deficiency and its association with glycemic control in children and adolescents with type 1 diabetes mellitus. J. Clin. Endocrinol. Metab..

[31] Barot K.S., Abbasi Z.A., Krishna Mohan G.V., Abid S.A., Hussain S.A., Wei C.R., Ali N. (2025). Prevalence of vitamin D deficiency in children and adolescents with type 1 diabetes mellitus: A systematic review and meta-analysis. Cureus.

[32] Chen C., Yang X., Li H., Sun Y., Wu L., Chang Y. (2024). Effect of vitamin D supplementation on glycemic control in type 1 diabetes mellitus: A meta-analysis. Clin. Nutr..

[33] Zipitis C.S., Akobeng A.K. (2008). Vitamin D supplementation in early childhood and risk of type 1 diabetes: A systematic review and meta-analysis. Arch. Dis. Child..

[34] Hyppönen E., Läärä E., Reunanen A., Järvelin M.R., Virtanen S.M. (2001). Intake of vitamin D and risk of type 1 diabetes: A birth-cohort study. Lancet.

[35] Littorin B., Blom P., Schölin A., Arnqvist H.J., Blohmé G., Bolinder J., Ekbom-Schnell A., Eriksson J.W., Gudbjörnsdottir S., Nyström L. (2006). Lower levels of plasma 25-hydroxyvitamin D among young adults at diagnosis of autoimmune type 1 diabetes compared with control subjects: Results from the nationwide Diabetes Incidence Study in Sweden (DISS). Diabetologia.

[36] Nascimento B.F., Moreira C.F.F., da Fonseca E.R., Fedeszen P.M.K., de Paula T.P., de Sena A.S.S., de Almeida N.F.A., Bandeira Filho O.C.S., Curval D.R., Padilha P.C. (2022). Effects of vitamin D supplementation on glycemic control of children and adolescents with type 1 diabetes mellitus: A systematic review. J. Pediatr. Endocrinol. Metab..

[37] Mathieu C., Gysemans C., Giulietti A., Bouillon R. (2005). Vitamin D and diabetes. Diabetologia.

[38] Pittas A.G., Dawson-Hughes B., Sheehan P.R., Ware J.H., Knowler W.C., Aroda V.R., Delahanty L.M., Barrett-Connor E., Crandall J.P., D2d Research Group (2019). Vitamin D supplementation prevention of type 2 diabetes. N. Engl. J. Med..

[39] Farahmand M.A., Daneshzad E., Fung T.T., Zahidi F., Muhammadi M., Bellissimo N., Azadbakht L. (2023). What is the impact of vitamin D supplementation on glycemic control in people with type-2 diabetes: A systematic review and meta-analysis. BMC Endocr. Disord..

[40] Cheng L., Lv C., Xue L., Zhang C., Wang L., Wang X., Chen S., Li X., Feng W., Xie H. (2024). The prevention and improvement effects of vitamin D on type 2 diabetes mellitus: Evidence from an umbrella review on meta-analyses of cohort studies and randomized controlled trials. Front. Nutr..

[41] Barbarawi M., Kheiri B., Zayed Y., Barbarawi O., Dhillon H., Swaid B., Yelangi A., Sundus S., Bachuwa G., Alkotob M.L. (2019). Vitamin D Supplementation and Cardiovascular Disease Risks in More Than 83 000 Individuals in 21 Randomized Clinical Trials: A Meta-analysis. JAMA Cardiol..

[42] Vojdeman F.J., Heegaard N.H.H., Madsbad S., Jørgensen M.E., Linneberg A., Andersen S., Thuesen B.H. (2022). Vitamin D deficiency and risk of type 2 diabetes: A cohort study. Diabetes Care.

[43] Papandreou D., Hamid Mehmood Z.-T.-N. (2016). An Updated Mini Review of Vitamin D and Obesity: Adipogenesis and Inflammation State. Open Access Maced. J. Med. Sci..

[44] Bouillon R., Marcocci C., Carmeliet G., Bikle D., White J.H., Dawson-Hughes B., Lips P., Munns C.F., Lazaretti-Castro M., Giustina A. (2019). Skeletal and extraskeletal actions of vitamin D: Current evidence and outstanding questions. Endocr. Rev..

[45] Poel Y., Hummel P., Lips P., Stam F., Van Der Ploeg T., Simsek S. (2012). Vitamin D and gestational diabetes: A systematic review and meta-analysis. Eur J Intern Med..

[46] Harvey N.C., Holroyd C., Ntani G., Javaid M.K., Cooper P., Moon R., Cole Z., Tinati T., Godfrey K., Dennison E. (2014). Vitamin D supplementation in pregnancy: A systematic review. Health Technol. Assess..

[47] Corcoy R., Mendoza L.C., Simmons D., Desoye G., Adelantado J.M., Chico A. (2020). The DALI vitamin D randomized controlled trial for gestational diabetes mellitus prevention: No major benefit shown besides vitamin D sufficiency. Clin. Nutr..

[48] Soheilykhah S., Mojibian M., Moghadam M.J., Jannati Moghadam M., Shojaoddiny-Ardekani A. (2013). The effect of different doses of vitamin D supplementation on insulin resistance during pregnancy. Gynecol. Endocrinol..

[49] Kron-Rodrigues M.R., de Souza A.I., Lima M.C., de Lima J.R., da Silva E.P., de Lima J.G. (2021). Supplementation of vitamin D in the postdelivery period of women diagnosed with previous gestational diabetes mellitus: A systematic review. Rev. Bras. De Ginecol. Obstetrícia.

[50] von Herrath M.G., Nepom G.T., Babu S.R., Atkinson M.A., Hummel P., Lips P., Simsek S. (2012). Is vitamin D deficiency involved in the pathogenesis of type 1 diabetes? A systematic review. Eur. J. Clin. Nutr..

[51] Ghosh S., Raj P., Mohapatra P.R. (2013). Vitamin D in type 1 diabetes mellitus: Current status and future prospects. Indian. J. Endocrinol. Metab..

[52] Karras S., Anagnostis P., Paschou S.A., Kandaraki E., Goulis D.G. (2015). Vitamin D status during pregnancy: Time for a more unified approach beyond borders?. Eur. J. Clin. Nutr..

[53] Pludowski P., Grant W.B., Karras S.N., Zittermann A., Pilz S. (2024). Vitamin D supplementation: A review of the evidence arguing for a daily dose of 2000 International Units (50 µg) of vitamin D for adults in the general population. Nutrients.

